# *N*-Feruloyl Serotonin Attenuates Neuronal Oxidative Stress and Apoptosis in Aβ_25–35_-Treated Human Neuroblastoma SH-SY5Y Cells

**DOI:** 10.3390/molecules28041610

**Published:** 2023-02-07

**Authors:** Meitong He, Chanhum Park, Yusu Shin, Jihyun Kim, Eunju Cho

**Affiliations:** 1 Department of Food Science and Nutrition, Kimchi Research Institute, Pusan National University, Busan 46241, Republic of Korea; 2 Institute of New Frontier Research Team, Research Institute of Medical-Bio Convergence, Hallym University, Chuncheon 24252, Republic of Korea; 3 Department of Medicinal Crop Research, National Institute of Horticultural and Herbal Science, Rural Development Administration, Eumseong 27709, Republic of Korea; 4 Department of Food Science and Nutrition, Gyeongsang National University, Jinju 52725, Republic of Korea

**Keywords:** amyloid beta, apoptosis, free radical, *N*-feruloyl serotonin, oxidative stress

## Abstract

Amyloid-beta (Aβ) aggregation and deposition have been identified as a critical feature in the pathology of Alzheimer’s disease (AD), with a series of functional alterations including neuronal oxidative stress and apoptosis. *N*-feruloyl serotonin (FS) is a plant-derived component that exerts antioxidant activity. This study investigated the protective effects of FS on Aβ_25–35_-treated neuronal damage by regulation of oxidative stress and apoptosis in human neuroblastoma SH-SY5Y cells. The radical scavenging activities increased with the concentration of FS, exhibiting in vitro antioxidant activity. The Aβ_25–35_-treated SH-SY5Y cells exerted neuronal cell injury by decreased cell viability and elevated reactive oxygen species, but that was recovered by FS treatment. In addition, treatment of FS increased anti-apoptotic factor B-cell lymphoma protein 2 (Bcl-2) and decreased the pro-apoptotic factor Bcl-2-associated X protein. The FS attenuated Aβ-stimulated neuronal apoptosis by regulations of mitogen-activated protein kinase signaling pathways. Moreover, activated CREB-BDNF signaling was observed by the treatment of FS in Aβ_25–35_-induced SH-SY5Y cells. These results demonstrate that FS shows potential neuroprotective effects on Aβ_25–35_-induced neuronal damage by attenuation of oxidative stress and apoptosis, and suggest that FS may be considered a promising candidate for the treatment of AD.

## 1. Introduction

Alzheimer’s disease (AD) is the most common type of dementia with features of amyloid-beta (Aβ) deposition, synaptic loss, and neuronal cell death, leading to cognition impairment [1]. High levels of Aβ induce oxidative stress, stimulate microglia/astrocyte activation, and cause apoptotic cell death in AD brains [2]. In addition, over-production of Aβ leads to the hyperphosphorylation of tau protein in the brain, which has indicated a relationship between Aβ pathway and tauopathy in AD pathology [1,3]. Previous studies have indicated that oxidative stress not only damages neuronal cells, but is also an early event in the pathology of AD [4]. In addition, with increasing age, overproduction of free radicals during aerobic respiration results in mitochondrial function disturbance, leading to oxidative damage that in turn promotes Aβ accumulation, which becomes a vicious cycle in the development of AD [5,6]. Therefore, Aβ-induced oxidative stress is closely related to the development of AD.

Oxidative stress induced by Aβ is involved in neuronal apoptosis of AD [7]. In intrinsic apoptosis pathway, B cell lymphoma-2 (Bcl-2) family members promote the permeability of mitochondrial membrane by decreasing Bcl-2 (an anti-apoptotic protein) and increasing Bcl-2-associated X protein (Bax, a pro-apoptotic protein) that release cytochrome c into cytoplasm [8]. Cytochrome c induces a caspase cascade that activates the cleavage of the death substrate poly-ADP-ribose polymerase (PARP) [9]. On the other hand, the mitogen-activated protein kinase (MAPKs) pathway including p38 mitogen-activated protein kinases (p38), c-Jun N-terminal kinase (JNK), and extracellular-regulated kinase (ERK) mediates apoptosis via the regulation of Bcl-2 proteins [10]. Numerous natural antioxidant compounds isolated from herbal medicines such as phenolic acids, flavonoids, alkaloids, and terpenoids have been demonstrated to exert anti-AD properties in Aβ-induced neuronal cell death [11,12,13].

**Figure 1:**
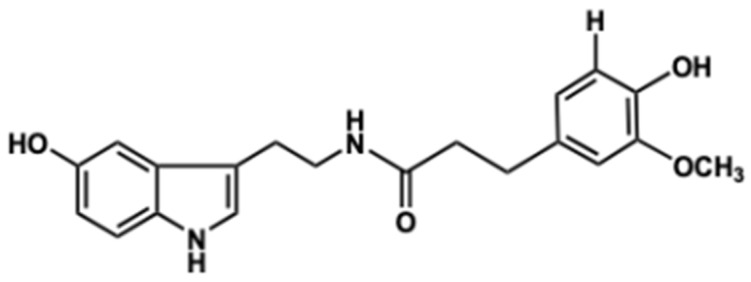
*N*-feruloyl serotonin.

*N*-feruloyl serotonin (FS; Figure 1) belongs to polyphenols [14], is a member of serotonin derivatives found in the seeds from safflower and *Leuzea carthamoides*, and cornflower [15,16,17]. Previous findings have revealed that FS exerts health beneficial effects such as anti-inflammation, antioxidant, and anti-apoptotic properties [18,19,20]. In particular, previous studies reported that FS decreased intracellular reactive oxygen species (ROS) and caspase activity in oxidative stress-induced neuronal cells [20,21]. However, neuroprotective effects and mechanisms of FS on neuronal oxidative stress and apoptosis induced by Aβ have yet to be clarified. Therefore, in the present study, we investigated the protective effects and mechanisms of FS on Aβ-induced neuronal oxidative stress and apoptosis in SH-SY5Y cells to determine the potential benefit of FS for AD prevention.

## 2. Results

### 2.1. In Vitro Free Radical Scavenging Activities

**Figure 2:**
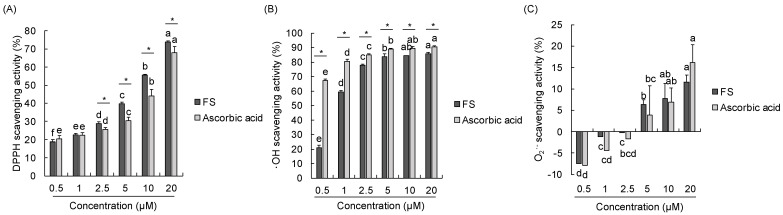
In vitro free radical scavenging activities of *N*-feruloyl serotonin. DPPH (**A**), •OH (**B**), and O_2_•^−^ (**C**) scavenging activities. Ascorbic acid as a standard. Values present means ± SD (*n* = 4). ^a–f^ Means with different letters are significantly different (*p* < 0.05) by Duncan’s multiple range test. * Represents significant difference (*p* < 0.05) between FS nad ascorbic acid. FS, *N*-feruloyl serotonin.

To identify the in vitro antioxidative activities of FS, we evaluated the DPPH, •OH, and O_2_•^−^ free radical scavenging activities of FS ranged from 0.5 to 20 µM as well as ascorbic acid as standard and summarized in Figure 2 and Table 1. The results showed that the DPPH radical scavenging activity increased with the increasing concentration of FS. The IC_50_ value of FS was 6.19 µM which was lower than the IC_50_ (9.19 µM) of ascorbic acid in DPPH radical scavenging activity. Moreover, •OH radical scavenging capacity of FS was increased with the increasing concentration and showed more than 80% from 5 µM. The IC_50_ was 1.35 µM for FS in •OH radical scavenging activity. The O_2_^−^ free radical scavenging activity showed that when the concentration increases up to 5 µM, the % scavenging turned into positive values both for FS and ascorbic acid.

### 2.2. Effects of FS on Cell Viability in Aβ_25–35_-Induced SH-SY5Y Cells

**Figure 3:**
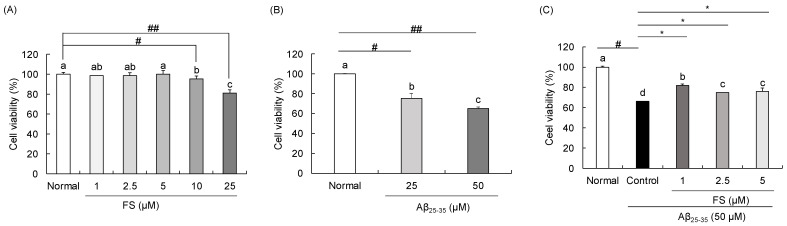
Effects of *N*-feruloyl serotonin on Aβ_25–35_-induced neuronal damage in SH-SY5Y cells. (**A**) Cell viability of FS (1, 2.5, 5, 10, 25 µM). (**B**) Cell viability of Aβ_25–35_ (25, 50 µM). (**C**) Cell viability of FS (1, 2.5, 5 µM) with Aβ_25–35_ (50 µM). Values present means ± SD (*n* = 5). ^a–d^ Means with different letters are significantly different (*p* < 0.05) by Duncan’s multiple range test. ^#^ *p* < 0.01, ^##^ *p* < 0.0001 vs. normal; * *p* < 0.05 vs. control. FS, *N*-feruloyl serotonin.

The FS ranged from 1 to 25 µM was first treated alone to SH-SY5Y cells (Figure 3A). No cytotoxicity was observed up to 5 µM, but significant cytotoxic effects over 10 µM. Therefore, FS at concentration of 1, 2.5, and 5 µM was used in the further experiments. In addition, we investigated the cell viability of Aβ_25–35_ at concentration of 25 and 50 µM. The result showed cell survival rate at 75.19% of 25 µM and 65.24% of 50 µM, respectively (Figure 3B). Therefore, we used 50 µM of Aβ_25–35_ in the further experiments for induce neuronal damage. Compared with the normal group (set as 100%), the cell toxicity increased after the treatment of Aβ_25–35_ at 50 µM, showing 66% of cell viability in the control group. However, FS at 1, 2.5, and 5 µM showed 82%, 75%, and 76% of cell viability, respectively. Therefore, treatment with FS plus Aβ_25–35_ significantly increased the cell viability compared with the controls that treated with Aβ_25–35_ alone (Figure 3C).

### 2.3. Effects of FS on Inhibition of Reactive Oxygen Species in Aβ_25–35_-Induced SH-SY5Y Cells

**Figure 4:**
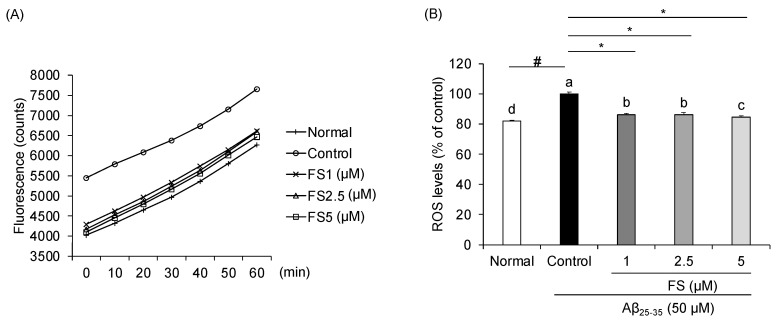
Effects of *N*-feruloyl serotonin on reactive oxygen species generation in Aβ_25–35_-induced SH-SY5Y cells. (**A**) Fluorescence of FS within 60 min. (**B**) ROS level of FS at 60 min. Values present means ± SD (*n* = 4). ^a–d^ Means with different letters are significantly different (*p* < 0.05) by Duncan’s multiple range test. ^#^ *p* < 0.01 vs. normal; * *p* < 0.05 vs. control. FS, *N*-feruloyl serotonin; ROS, reactive oxygen species.

To investigate whether Aβ_25–35_-induced toxicity was related to oxidative stress, the ROS level was measured. When compared with the normal cells, Aβ_25–35_ significantly increased the intracellular ROS level within 60 min in time dependent manner and was reduced by FS (Figure 4A). Moreover, at 60 min, the % ROS levels significantly increased after the treatment of Aβ_25–35_ in SH-SY5Y cells. However, % ROS levels were significantly decreased by treatment of FS (1, 2.5, and 5 µM) at 86%, 86%, and 84%, respectively, compared with Aβ_25–35_-treated control group (Figure 4B).

### 2.4. Effects of FS on Apoptosis-Related Protein Expression in Aβ_25–35_-Induced SH-SY5Y Cells

**Figure 5:**
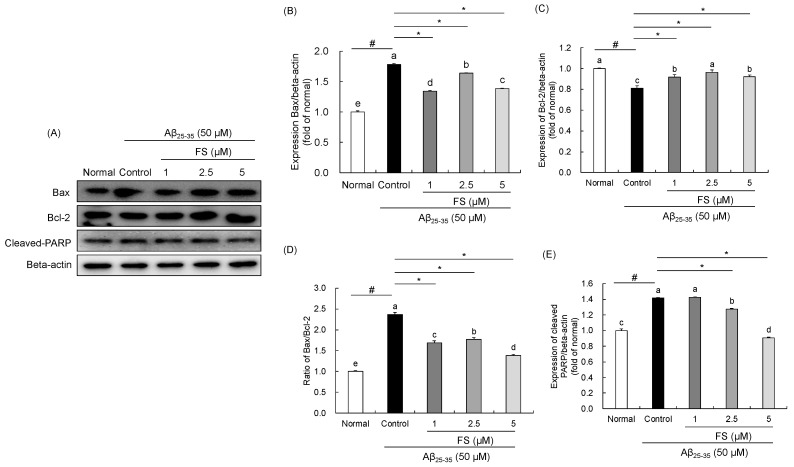
Effects of *N*-feruloyl serotonin on expressions of Bax, Bcl-2, and cleaved PARP in SH-SY5Y cells. The western blot bands (**A**), relative protein levels of Bax (**B**), Bcl-2 (**C**), ratio of Bax and Bcl-2 (**D**), and cleaved PARP (**E**). Values present means ± SD (*n* = 3). ^a–e^ Means with different letters are significantly different (*p* < 0.05) by Duncan’s multiple range test. ^#^ *p* < 0.01 vs. normal; * *p* < 0.05 vs. control. FS, *N*-feruloyl serotonin.

We examined the protein expressions of Bax, Bcl-2, and cleaved PARP which are regarded as the markers to the apoptosis pathway (Figure 5). The Aβ_25–35_ significantly induced apoptosis by up-regulations of Bax protein expression and down-regulation of Bcl-2 in SH-SY5Y cells. However, in the groups treated with FS at 1, 2.5, and 5 µM, the expression of Bax down-regulated, whereas Bcl-2 was up-regulated. Moreover, quantification of Bax/Bcl-2 level in control group significantly increased, showing 2.37-fold of the normal group, and that was reversed by the treatment of FS (1, 2.5, 5 µM). In addition, the protein expression of cleaved PARP (1.42 ± 0.01, *p* < 0.05) was up-regulated after the treatment Aβ_25–35_ when compared to the normal cells; however, it was reversed by FS. These results supported the anti-apoptotic effects of FS on Aβ_25–35_-induced SH-SY5Y cells.

### 2.5. Effects of FS on Activation of MAPKs Signaling Pathway in Aβ_25–35_-Induced SH-SY5Y Cells

**Figure 6:**
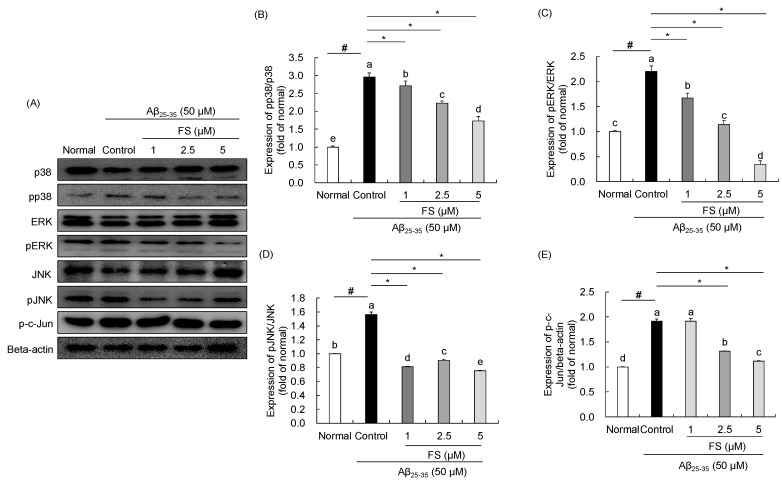
Effects of *N*-feruloyl serotonin on MAPKs signaling pathway in SH-SY5Y cells. The western blot bands (**A**), relative protein levels of pp38/p38 (**B**), pERK/ERK (**C**), pJNK/JNK (**D**), and p-c-Jun (**E**). Values present means ± SD (*n* = 3). ^a–e^ Means with different letters are significantly different (*p* < 0.05) by Duncan’s multiple range test. ^#^ *p* < 0.01 vs. normal; * *p* < 0.05 vs. control. FS, *N*-feruloyl serotonin.

As shown in Figure 6, treatment of 50 µM Aβ_25–35_ significantly up-regulated the protein expressions of phosphorylated p38 (2.96 ± 0.12, *p* < 0.05), ERK (2.20 ± 0.12, *p* < 0.05), and JNK (1.56 ± 0.04, *p* < 0.05). Compared to the protein expression in the control group, treatment with 1, 2.5, and 5 µM of FS significantly down-regulated the pp38, pERK, and pJNK in a concentration dependent manner except for the expression of pJNK. The Aβ_25–35_-treated control group showed a significant 1.91-fold increase of p-c-Jun protein expression compared with the normal group, and that was suppressed by the treatment of FS. In particular, the treatment with 2.5 and 5 µM of FS led to significant decreased protein expressions of p-c-Jun compared with control group.

### 2.6. Effects of FS on the Regulation of CREB-BDNF Signaling in Aβ_25–35_-Induced SH-SY5Y Cells

**Figure 7:**
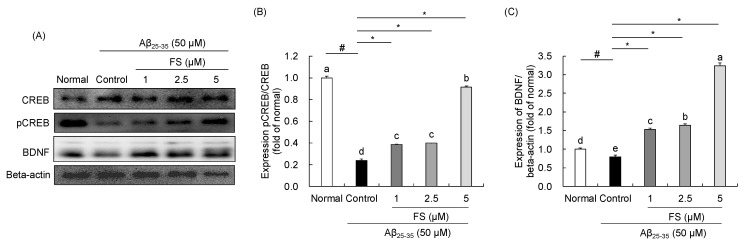
Effects of *N*-feruloyl serotonin on the regulation of CREB-BDNF signaling in SH-SY5Y cells. The western blot bands (**A**), relative protein levels of pCREB/CREB (**B**) and BDNF (**C**). Values present means ± SD (*n* = 3). ^a–e^ Means with different letters are significantly different (*p* < 0.05) by Duncan’s multiple range test. ^#^ *p* < 0.01 vs. normal; * *p* < 0.05 vs. control. FS, *N*-feruloyl serotonin.

As shown in Figure 7, the protein expressions of CREB and BDNF were measured. In Aβ_25–35_-treated control group, decreased of phospho-CREB (−0.24 ± 0.01, *p* < 0.05) and BDNF (−0.80 ± 0.04, *p* < 0.05) protein expressions were showed when compared with normal group. However, FS at concentration of 1, 2.5, and 5 µM increased the levels of these proteins compared with Aβ_25–35_-treated control group. In particular, 5 µM FS led to high increase in pCREB/CREB (0.92 ± 0.01, *p* < 0.05) and BDNF (3.23 ± 0.08, *p* < 0.05) expressions.

## 3. Discussion

The world’s old population (over aged 65) was over 700 million in 2019 and it was estimated to be doubled to 1.5 billion by 2050 [22]. The rapid size and proportion of aging society increases the prevalence of neurodegenerative diseases such as dementia. In 2020, more than 50 million people are living with dementia worldwide which aggravates the social and economic burden [23]. Up to now, the investigation to identify the mechanisms of AD pathology is still in process. Various therapies that target AD hypotheses have undergone preclinical and clinical studies [24]. However, they do not succeed to be continued in the late-stage human trials due to the low bioavailability and half-life of the drugs, and side-effects on the trial subjects [25]. Before the appearance of modern medicine, herbal medicines occupied incomparable advantages in complex diseases therapeutic approach because of the multiple biological targets of their active ingredients [26].

The etiology and pathogenesis of AD are complex, and the disease is irreversible, which has become a public health problem by its both direct and indirect impacts [1]. Aβ toxicity is well-known in the research of AD and many therapeutic approaches have been proposed based on the amyloid cascade hypothesis [2]. It has been indicated that Aβ deposits are found in large amounts in the brains of AD patients which are suggested as the critical events in the origin and progression of neuronal damage [27]. Previous studies have highlighted that exogenous Aβ treatment can effectively serve as a model for a toxic mechanism of synaptic dysfunction, mitochondrial alteration, oxidative stress, and apoptosis both in vitro and in vivo [28,29]. The SH-SY5Y neuroblastoma cell line has been frequently used as an in vitro model for neurodegenerative disease studies such as AD [30]. Exposure to Aβ for 24 h markedly induced morphological changes in SH-SY5Y cells with the reduction in cell numbers and cell body shrinkage [31]. Therefore, we established the Aβ-mediated toxicity in SH-SY5Y cells as a suitable AD cell model in the present study.

FS is one of the major serotonin derivatives and the most abundant polyphenol in *C. tinctorius* L. seeds, exhibiting health benefits against neurodegenerative disorders, cardiovascular disease, and diabetes [32]. Chemically, FS is an indole hydroxycinnamic acid amide that synthesized by serotonin *N*-(hydroxycinnamoyl)transferase [21,33]. Serotonin derivatives are mostly found in the plant seeds, with contents ranging from 0.1 to 740 µg/g [33]. Previously, our research team determined the memory protective effect of *C. tinctorius* L. seeds extract against scopolamine in mice and identified that the content of FS at 5.57 mg/kg [34]. FS has antioxidant property against oxidative-related diseases by reducing the level of low-density lipoproteins in atherosclerotic development [35]. FS also has been reported to decrease oxidative stress-mediated inflammatory responses by inhibiting MAPKs and NF-ĸB signaling pathways in cisplatin-inflicted mouse model [18]. Moreover, protective effect of FS on neuronal damage by inhibiting the activation of cascade-3 has been demonstrated [21]. However, neuroprotective mechanism of FS on Aβ-induced neuronal cell damage is not yet clear. Therefore, in the present study, we demonstrated the neuroprotective effects and mechanisms via the regulation of oxidative stress, neuronal apoptosis, and CREB/BDNF signaling in Aβ_25–35_-induced SH-SY5Y cells.

Free radicals generated constantly during aerobic respiration and can be scavenged by the antioxidation system in the body [36]. When this system cannot maintain the balance between anti-oxidation and pro-oxidation, oxidative damage towards the body occurs. Free radical generation is directly related to oxidation. Therefore, several methods have been reported to determine the free radical scavenging activities. The DPPH free radical assay is widely used for quick testing in antioxidant activities of plant extracts, foods, and natural products [37]. DPPH is a stable free radical in purple color, which is changed into yellow when a substance with antioxidant ability that can donate a hydrogen atom [38]. In the present study, DPPH free radical scavenging activity of FS is concentration dependent as reflecting in the increase concentration of FS, the increase of free radical scavenging activity. In addition, IC_50_ value is the concentration of sample which has ability to scavenge 50% of the free radicals; thus, the lower IC_50_ value the higher antioxidant activity [39]. The IC_50_ (6.19 µM) of FS in DPPH radical scavenging was lower than the standard antioxidant ascorbic acid (IC_50_ = 9.19 µM). As mentioned, the ability of DPPH free radical scavenging is based on donating electrons (hydrogen atoms) that is the higher DPPH scavenging by more hydrogen donors. In structure-related activity, the antioxidant ability of FS could be attributed to the two hydroxyl groups in the aromatic ring and the presence of phenolic amide and serotonin moiety, which support the earlier reports [40,41]. •OH is known to be the most active free radical and it can be generated in vitro in the presence of iron ions and hydrogen peroxide according to the Fenton reaction [42]. In this study, FS showed low IC_50_ value and high antioxidant effect (over 80%) at concentration of 5 µM, which support the previous study that strong antioxidant efficiency can be enhanced by phenolic rings and the increase of methoxy group of the compound [42,43]. Moreover, O_2_•^−^ free radical is a major ROS in living organisms which can be scavenged by the antioxidant enzymes such as superoxide dismutase [42]. A previous study has demonstrated that FS did not show significant regulated effect on the protein expression of superoxide dismutase in vivo [18]. Likewise, in the present study, we found positive effects of both FS and ascorbic acid in O_2_•^−^ radical scavenging activity until 5 µM, indicating that FS exhibits minor ability on O_2_•^−^ free radical scavenging.

Oxidative stress is reflected in the intracellular levels of ROS production follow by damage to lipids, proteins, and DNA [7]. Evidence showed that Aβ stimulation results in cytotoxicity and promotes ROS generation which leads to disrupting blood-brain barrier integrity [44]. In addition, previous studies showed clear changes in morphology and resulted in about 40% of cell death at 50 µM after the addition of Aβ_25–35_ for 24 h [45,46]. Therefore, to induce the neuronal cell damage, we used 50 µM of Aβ_25–35_ in this study. In the present study, we observed that treatment with FS at 1, 2.5, and 5 µM significantly decreased the loss of cell viability induced by Aβ in SH-SY5Y cells. To understand the protective mechanism of Aβ-induced oxidative stress, the ROS level was measured. Overloaded ROS is the main factor to damage the anti-oxidation system in the body [47]. In addition to the suppression of ROS production, inhibiting oxidative stress by enhancing the antioxidant enzymes such as SOD, CAT, and GPX is also a strategy in neuroprotective therapy [48]. Several studies reported the effects of FS on oxidative stress-related markers. It has been demonstrated that administration of FS significantly increased the antioxidant protein levels of GPx in cisplatin-induced renal damaged mice, but no differences in the levels of SOD and CAT [18]. A previous study has reported that treatment of FS significantly decreased the four-fold-increasing mitochondrial superoxide induced by high glucose in PC12 cells, indicating that FS effectively inhibits high glucose-induced mitochondrial dysfunction by reducing intracellular ROS levels [21]. In addition, ROS are mainly generated from mitochondria that can trigger the changes of mitochondrial membrane potential [49]. Supplemented with FS attenuated lipid peroxidation by approximately 50% decreased of TBARS formation in apoE-deficient mice plasma [35]. In this study, we confirmed that the level of ROS significantly elevated by Aβ_25–35_ treatment, which may also be attributed to apoptotic cell death. However, FS showed an inhibitory effect on ROS production concentration-dependently, suggesting that FS is beneficial on Aβ-induced oxidation stress. These results indicated that FS may protect against Aβ-induced oxidative stress by inhibiting ROS generation.

The mitochondria are mainly responsible for over 90% of ROS production [50]. Elevated ROS levels reflect apoptotic cell death via the regulation of Bcl-2 family proteins, the pro-apoptotic protein (Bax) and the anti-apoptotic protein (Bcl-2) [51]. In addition, the ratio between Bax and Bcl-2 is known to determine the susceptibility to apoptosis [51]. The ratio of Bax/Bcl-2 also has mentioned as a rheostat in regulating the mitochondrial function [52]. Increased ratio of Bax to Bcl-2 promotes the mitochondrial membrane permeability and that leads to the release of cytochrome c and directly activates caspase cascade [53]. PARP cleavage by caspase has considered a hallmark of apoptosis and has been implicated in AD [54]. A previous study has reported that treatment of FS significantly reduced apoptotic cell death in high glucose-treated neuronal cells. In particular, the flow cytometry analysis showed a significant inhibitory effect of apoptosis while compared with the high glucose treated PC12 neuronal cells [21]. Moreover, treatment of FS significantly decreased apoptotic cell ratio to 14.2% in LPS-induced apoptosis of HaCaT cells via annexin V/propidium iodide staining [55]. We measured the protein expressions of Bax, Bcl-2, and cleaved PARP in Aβ-induced cell damage. FS has effect on down-regulating Bax and cleaved PARP and up-regulating Bcl-2, as well as reducing the Bax to bcl-2 ratio in Aβ_25–35_-stimulated SH-SY5Y cells, showing an improved effect of FS on Aβ-induced mitochondrial dysfunction. However, the further study on protective effects of FS on mitochondria dysfunction by using for instance, flow cytometry analysis in Aβ-induced neuronal cells that is considered necessary.

In addition to mitochondrial pathway, activation of MAPKs signaling pathway including p38, JNK, and ERK is associated with cell proliferation, oxidative stress, inflammatory responses, and apoptosis [56]. p38, ERK, and JNK are the representative proteins in the regulation of MAPKs signaling and their phosphorylated forms confluences with apoptosis pathway which can balance cell death and survival [57]. Activation of p38, JNK, and ERK increased Bax expression and decreased Bcl-2 expression to induce apoptosis [58]. We determined the role of FS on the activation of MAPKs signaling pathway in Aβ_25–35_-induced SH-SY5Y cells. Consistent with the previous studies [58,59], results on protein expressions demonstrated that phosphorylation of p38, ERK, and JNK were increased in Aβ_25–35_-induced cell damage and were suppressed by FS treatment in SH-SY5Y cells. Phosphorylated c-Jun is the downstream target of JNK, which can be increased by the increase of pJNK transcription and translation [60]. c-Jun phosphorylation by JNK is necessary for apoptotic response to promote neuronal cell death [60]. We found that significant downregulated p-c-Jun expression by FS in Aβ_25–35_-induced SH-SY5Y cells. These suggest that FS treatment reduce apoptosis in SH-SY5Y cells via regulation of MAPKs signaling pathway.

**Figure 8:**
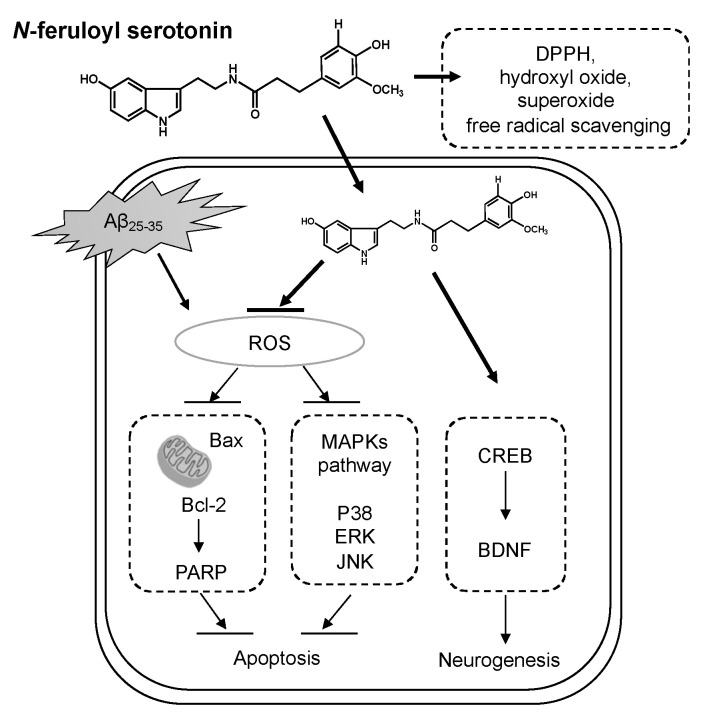
Summary of the action of *N*-feruloyl serotonin on Aβ-induced neuronal damage.

The CREB and BDNF are well-known for neuronal development and cell survival, and synaptic plasticity [61]. Cumulative studies have suggested that Aβ mediated by its toxic effect on neurotrophic factor expression may lead to neurodegeneration [62]. BDNF, a typical neurotrophic factor and a small dimeric protein, which is involved in neuronal survival and promotes hippocampal neurogenesis in the region of dentate gyrus [63,64]. It has been revealed that BDNF protects neuronal cells from oxidative stress-induced cell injury [65]. Additionally, CREB is a transcription factor and regulates BDNF transcription [61]. Consistent with the previous studies [65], we observed that phosphorylated CREB and BDNF protein expressions under Aβ_25–35_ stimulation were reduced in SH-SY5Y cells. It has been reported that phenolic compounds such tea polyphenols and pterostilbene were able to pass through the blood-brain barrier and modulate the CREB-BDNF signaling pathway, leading to synaptic junction recovery [65,66]. In the present study, the treatment with FS effectively recovered Aβ_25–35_-induced decrease in pCREB and BDNF expressions, suggesting that FS might potentially attenuate neuronal damage via activating CREB-BDNF signaling pathway (Figure 8).

There are several limitations in the present study. First, to development of prevention and treatment materials in AD, further studies are needed to focus on the neuroprotective effects of FS under in vivo system such as animal and clinical studies. Previous studies have demonstrated beneficial effects of FS such as anti-inflammatory effect and anti-atopic dermatitis under in vivo system [17,67]. However, in the current stage, lack of enough data to support the neuroprotective effect of FS under in vivo system. Therefore, neuroprotective effects of FS under in vivo and human studies are needed to extend the further research such as toxicity test, pharmacological test, and mechanism research on animal studies. In addition, to examine the cytotoxicity of FS under clinical studies, study on the identify the acceptable daily intake of FS needed. Moreover, as the pathology of AD is multi-direction, the mechanism studies of the well-known features of AD, such as Aβ generation and tau protein aggregation are necessary. In particular, the SH-SY5Y cells are easy handling and are commonly used as a neuron-like cell model in the areas of neuroscience research, especially the AD. Therefore, we used SH-SY5Y cells as a cell model of AD in the present study. However, due to the pathology of AD is complex, strategies for the prevention and treatment of AD need the multi-targeting and multi-direction. Therefore, to understand more detailed about the mechanism of AD, in our further study related to neuroprotective effect, we will use other cell lines, such as microglial cells or astrocyte, as cell models of AD.

## 4. Materials and Methods

### 4.1. Chemicals

*N*-feruloyl serotonin (FS) was purchased from Santa Cruz Biotechnology, Inc. (SC-498142; Dallas, TX, USA). 1,1-Dephenyl-2-picrylhydrazyl (DPPH), 2-deoxyribose, Aβ_25–35_ (A4559), and 2′,7′-dichlorofluorescein diacetate (DCF-DA) were obtained from Sigma-Aldrich Inc. (St. Louis, MO, USA). 3-(4,5-Dimethylthiazol-2-yl)-2,3-diphenyl tetrazolium bromide (MTT), phenazine methosulfate (PMS), NADH disodium salt, and nitrotetrazolium blue chloride (NBT) were purchased from Bio Basic Inc. (Toronto, Ontario, Canada). Dulbecco’s Modified Eagle’s Medium (DMEM), fetal bovine serum (FBS), penicillin-streptomycin solutions, and trypsin-EDTA solution were purchased from Welgene Inc. (Daegu, Republic of Korea). Dimethyl sulfoxide (DMSO) and FeSO_4_·7H_2_O were obtained from Daejung Chemicals & Metals Co., Ltd. (Siheung-si, Republic of Korea). Hydroxyl peroxide (H_2_O_2_) was purchased from Junsei (Tokyo, Japan). Thiobarbituric acid (TBA) and trichloroacetic acid (TCA) were obtained from Acros Organics (Fair Lawn, NJ, USA) and Kanto Chemical Co. Inc. (Tokyo, Japan), respectively. Radio-immuno-precipitationassay (RIPA) buffer was purchased from Elpics Biotech (Daejeon, Republic of Korea), protease inhibitor cocktail was purchased from Calbiochem (Cambridge, MA, USA), and polyvinylidene fluoride (PVDF) membrane was obtained from Millipore (Bedford, MA, USA). Primary and secondary antibodies (Bax, #2772; Bcl-2, ab196495; PARP, #9532; p38, #9212; pp38, #9211; ERK, #4695; pERK, #4370; JNK, #9252; pJNK, #4668; p-c-Jun, #3270; CREB, #9197; pCREB, #9198; BDNF, ab108319; beta-actin, #8457; anti-rabbit IgG, HRP-linked antibody, #7074) were purchased from Cell Signaling Technology, Inc. (Danvers, MA, USA) and Abcam (Cambridge, UK).

### 4.2. Measurement of Free Radical Scavenging Activities

#### 4.2.1. 1,1-Dephenyl-2-picrylhydrazyl (DPPH) Assay

The DPPH radical scavenging activity was measured according to the procedure described by Suja et al. [68], with modifications. An ethanolic solution of DPPH radical at 60 µM was freshly prepared. The 100 µL of FS (0.5–20 µM) as well as the standard compound (ascorbic acid; 0.5–20 µM) in DMSO were added to 100 µL of DPPH solution, using ethanol as blank at room temperature and mixed for 30 min. Absorbance was measured at 517 nm using a microplate reader (Rayto Life and Analytical Sciences Co., Ltd., Shenzhen, China). The IC_50_ value was calculated. The DPPH free radical scavenging activity was calculated using the following formula:

% Scavenging activity = [Abs_control_ − Abs_sample_/Abs_control_] × 100

#### 4.2.2. Hydroxyl Radical (•OH) Assay

The •OH scavenging potential of FS was measured according to the method of Klein et al. [69], with modifications. Briefly, each concentration of FS (0.5–20 µM), as well as ascorbic acid (0.5–20 µM), were mixed with 10 mM FeSO_4_·7H_2_O-EDTA, 10 mM 2-deoxyribose, and 10 mM H_2_O_2_, and incubated at 37 °C for 4 h. Then 1% TBA solution and 2.8% TCA solution were added to the mixture and water bath (100 °C) for 20 min. Absorbance was measured at 490 nm using a microplate reader. The inhibition percentage for scavenging •OH radical and IC_50_ value was calculated.

#### 4.2.3. Superoxide (O_2_•^−^) Assay

The method of O_2_•^−^ radical scavenging was referred to Nishikimi et al. [70], with modifications. The FS (0.5–20 µM) and ascorbic acid (0.5–20 µM) in diluted water were mixed with 0.1 M Tris-HCl (pH 7.4), 100 µM PMS, 500 µM NBT, and 500 µM NADH. And then, the mixtures were incubated at room temperature for 10 min and measured at 560 nm using a microplate reader. The inhibition percentage for scavenging O_2_•^−^ radical and IC_50_ value were calculated.

### 4.3. Cell Culture

The SH-SY5Y neuroblastoma cell line (CRL-2266) was purchased from the American Type Culture Collection (Manassas, VA, USA) and cultured in DMEM supplemented with 10% FBS and 1% penicillin-streptomycin at 37 °C in 5% CO_2_ atmosphere. Cells were seeded at a density of 2.5 × 10^5^ and 1 × 10^6^ cells/mL in 96- and 6-well plate, respectively. FS was diluted with cell culture medium for use. The Aβ_25–35_ was dissolved in sterilized distilled water and incubated at 37 °C for 72 h and then diluted with cell culture medium at concentration of 50 µM prior to use.

### 4.4. Measurement of Cell Viability

Cell viability was measured using a MTT assay [71]. Cells were seeded at 2.5 × 10^5^ cells/mL in a 96-well plate. FS was treated at different concentrations for 4 h, and then Aβ_25–35_ (50 µM) was added in all groups except the normal group. MTT solution (5 mg/mL) was added after 24 h and the formazan was dissolved in DMSO. The absorbance at 540 nm was read using a microplate reader.

### 4.5. Measurement of ROS Production

The ROS level was measured using DCF-DA dye [72]. Cells (2.5 × 10^5^ cells/mL) were seeded a in a black 96-well plate. FS (1, 2.5, 5 µM) was treated at different concentrations followed with Aβ_25–35_ (50 µM) treated in all groups except the normal group. DCF-DA solution was added and incubated for 30 min. The fluorescence intensity was read using a microplate reader for fluorescence (BMG Labtech, Ortenberg, Germany) at an excitation wavelength of 485 nm and emission wavelength of 520 nm.

### 4.6. Measurement of Protein Expressions

Cells were seeded at 1 × 10^6^ cells/mL in 6-well plate for 24 h, then treated with FS followed with Aβ_25–35_ treated in all groups except the normal group. For protein expression analysis, the cells were lysed with lysis buffer (1× protease inhibitor cocktail and RIPA buffer) and the proteins were isolated. The equal amount of protein sample was separated on 8–13% polyacrylamide gels by electrophoresis, and then transferred to polyvinylidene difluoride membranes. The membranes were incubated with 5% skim milk to block non-specific binding, and subsequently incubated with primary antibodies (Bax, 1:1000; Bcl-2, 1:500; PARP, 1:1000; p38, 1:1000; pp38, 1:1000; JNK, 1:1000; pJNK, 1:200; ERK, 1:1000; pERK, 1:500; p-c-Jun, 1:1000; CREB, 1:500; pCREB, 1:500; BDNF, 1:500; beta-actin, 1:1000) overnight at 4 °C. The membranes were incubated with correlated secondary antibody (anti-rabbit IgG, HRP, 1:1000) then exposed to enhanced chemiluminescence solution and detected using chemiluminescent detection system (Davinch Chemi^TM^, Seoul, Republic of Korea). Detected bands were analyzed using ImageJ^®^ software (v1.53, NIH, Bethesda, MD, USA).

### 4.7. Statistical Analysis

Data were expressed as mean ± standard deviation (SD). One-way analysis of variance (ANOVA) followed by Duncan’s multiple test (SPSS 26.0, SPSS Inc., Chicago, IL, USA) were used for statistical analysis. Significant differences were determined as *p* < 0.05.

## 5. Conclusions

In conclusion, the present study demonstrated that FS showed antioxidant properties on free radical scavenging in vitro. Treatment with FS could attenuate Aβ-induced oxidative stress via inhibiting ROS production, attenuating apoptosis by regulation of Bax/Bcl-2 and MAPKs, and activating CREB-BDNF neurogenesis pathway in SH-SY5Y cells (Figure 8). Therefore, FS might serve as a promising candidate to combat oxidative stress-related neurodegenerative diseases such as AD.

## Figures and Tables

**Table 1:** In vitro free radical scavenging activities of *N*-feruloyl serotonin in IC_50_ value (µM).

	DPPH	•OH	O_2_•^−^
FS	6.19 ± 0.09	1.35 ± 0.03	>100
Ascorbic acid	9.19 ± 0.69	0.03 ± 0.00	>100

Values present means ± SD. IC_50_, half-maximal inhibitory concentration; FS, *N*-feruloyl serotonin.

## Data Availability

Data is contained within the article.

## Sample Availability

Samples of the compounds are not available from the authors.
